# Supplementary material for: A gravity-driven sintering method to fabricate geometrically complex compact piezoceramics
Source: Nat Commun. 2021 Oct 18;12:6066. doi: 10.1038/s41467-021-26373-x (PMC8523652; doi:10.1038/s41467-021-26373-x)
Supplement: Supplementary file 1 — Supplementary Information [file 41467_2021_26373_MOESM1_ESM.pdf]

Supplementary Materials for

A gravity-driven sintering method to fabricate geometrically complex compact piezoceramics

Supplementary Note 1 | Explanation of the mechanical measurement

The mechanical properties of ceramics are important for practical applications. Tensile strength is usually used to evaluate the mechanical quality of ceramic specimens. Normally, to avoid fracture at the gripping area, the testing specimens are designed and fabricated in the dog-bone shape [47],[48]. However, difficulties in fabricating the dog-bone piezoceramic specimens hinder conducting tensile tests on piezoceramics. To solve this problem, we designed four tapered tabs to clamp the PZT slices as schematically shown in Supplementary Figure 4a-b. The PZT slices are bonded on the glass-fiber tabs, and then the testing specimens are clamped by the tensile machine (Supplementary Figure 4c). It should be mentioned here that the clamping strategy used in this work can effectively prevent the testing PZT slices from fracture during the initial clamping state as the clamping forces are directly applied on the four tabs. The force-displacement curves are shown in Supplementary Figure 4d, where the insets exhibit the fracture of the PZT slices. Although this special clamping method causes high measured deformation, the tensile strengths of the testing specimens are comparable with the recorded values (around 40~50 MPa) as the fracture caused by the tensile force occurs at the middle of the PZT slice (inset in Supplementary Figure 4d).

Supplementary Note 2 | Explanation of the tuneability

In the simplified model shown in Fig. 3d, the gravitational force $\mathbf{F}_g$ is balanced by the equivalent resistance force $\mathbf{F}_r$, and the equilibrium can be written as:

$$\mathbf{F}_g = 2\cos\theta\mathbf{F}_r \quad (1)$$

For the three tuning regions, we consider a small deformation where the slight change of configuration has a negligible effect on θ ($\cos\theta$). To further study the tuning abilities of the three regions, we consider the configuration evaluation from the view of forming new equilibrium, which is determined by $\mathbf{F}_g$, $\mathbf{F}_r$ and θ. The range of θ for each tuning region is shown in Fig. 3b and the corresponding value of $\cos\theta$ is listed in Supplementary Figure 6. We first discuss the tunability of the two LT regions. As illustrated in the main text, in region B (Fig. 3b), the small variation of $\mathbf{F}_g$ requires a large change of $\mathbf{F}_r$ due to low $\cos\theta$ (Supplementary Figure 6). This means that a slight modification of the configuration comes with a dramatic change of the designing parameter (effective length l_e). For region D, the value of $2\cos\theta$ range from 0.584 to 1.06, which indicates that the configuration is highly sensitive to the designing parameter. In the OT region, the moderate value of $2\cos\theta$ enables a relatively stable tuning of the configuration.

Supplementary Note 3 | Explanation of parameters with * superscipt in Table 1

Scalability for scale-up production. In practical 3D printing ceramic process, the printer normally produces only one specimen within one processing cycle. This means that to fabricate *N* piezoceramic green bodies, one printer has to run *N* times processing cycle. Furthermore, the high resolution of 3D printing processes usually requires low printing speed, which predestines them to exhibit low scalability in scale-up production.

In CTE-based methods, the curvatures of fabricated specimens are tuned via modifying the thickness of each layer and heating setup (e.g. heating rate and temperature). This fabrication characteristic allows users simultaneously process several specimens with similar laminate structures under the same

heating setup. In the pre-stressed method, the curvatures of post-processing piezoceramics are strongly dependent on the particular laminate structures of the specimens. Similarly, only specimens that have the same laminate design can be simultaneously strained by one fabrication device. The discussion above illustrates that a given laminate structure corresponds to a particular processing setup (heating and loading setup) that is fixed during one processing cycle. Therefore, the two post-processing methods are not available for scalable production of curved piezoceramics with diverse geometries.

We demonstrated the potential of the GDS process for massive production in the main text and the corresponding schematic is shown in Fig. 4a. As a demonstration of this scalability, we fabricated 24 PZT ceramics with 3 different configurations in one GDS processing cycle. Our GDS process exhibits a good feasibility for large-scaled production without any restrictions on the geometrical similarity of the processing specimens. Based on the elaboration above, we confirm that our GDS process is superior than 3D-printing techniques and post-processing methods currently available from the view of scale-up production.

Compactness [piezoceramic ratio in green bodies]. Compactness is defined as the ratio of piezoceramic constituent in the green body. For example, in the GDS process, the commercial PZT powders are mixed with PVA binder and then they are pressed to a rectangle shape. Hence the compactness of the PZT green body used in our GDS process is measured as 99 vol%.

For slurry-based 3D-printing technologies, the high ratio of piezoceramic constituent in the ceramic/polymer mixture usually results in a high viscosity of feedstock, which deteriorates their ability to print complex-geometry piezoceramic green bodies. For bulk solid-based methods, the composite filaments are prepared by loading piezoceramic particles into thermoplastic binders. Similar to slurry-based methods, the fused piezoceramic/polymer filaments comprised a high ratio of piezoceramic particles that undermines the processibility of feedstock. Powder-based ceramic 3D printing technologies are mainly used for processing porous ceramic components as the powder beds containing loose ceramic particles work as feedstock. It should be noted that the compactness listed in Table 1 are the highest ratio mentioned among the reference works that focus on studying 3D printing of piezoelectric materials.

Geometrical limits. The GDS method are not suitable for fabricating piezoceramics in over than half-round arches and close-loop shapes as the main driven force using in this method is gravitational force. In addition, the GDS method take advantage of the thermal-induced creep effect for shaping, so the final geometry of the GDS-fabricated piezoceramic is limited by the surface tension of the liquid-like semifinal specimen for example square and cone shapes.

Piezoelectricity [d_{33} pC/N]. Piezoelectricity is an important feature of piezoceramics, which enables conversion between electrical and mechanical signals. We use the piezoelectric coefficient d_{33} to characterize the piezoelectricity of the piezoceramics discussed in Table 1. The post processing methods directly process compact piezoceramic sintered bodies, therefore, the value of d_{33} is mainly determined by the piezoelectricity of the used piezoceramic layer. The differences in d_{33} between the GDS-fabricated and post-processing curved ceramics originate from the material composition of the purchased PZT material. However, for 3D printing methods, low volume of piezoceramic constituents in the printed green bodies predestine the as-sintered piezoceramics in porous bodies and thus compromised piezoelectricity (small value of d_{33}) compared with the other two kinds of methods.

Supplementary Figures

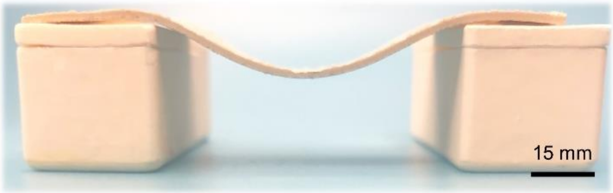

Supplementary Figure 1 | Photograph of GDS-sintered ceramic. The obtained curved PZT ceramic is corresponding to the sample recorded in Fig. 2a.

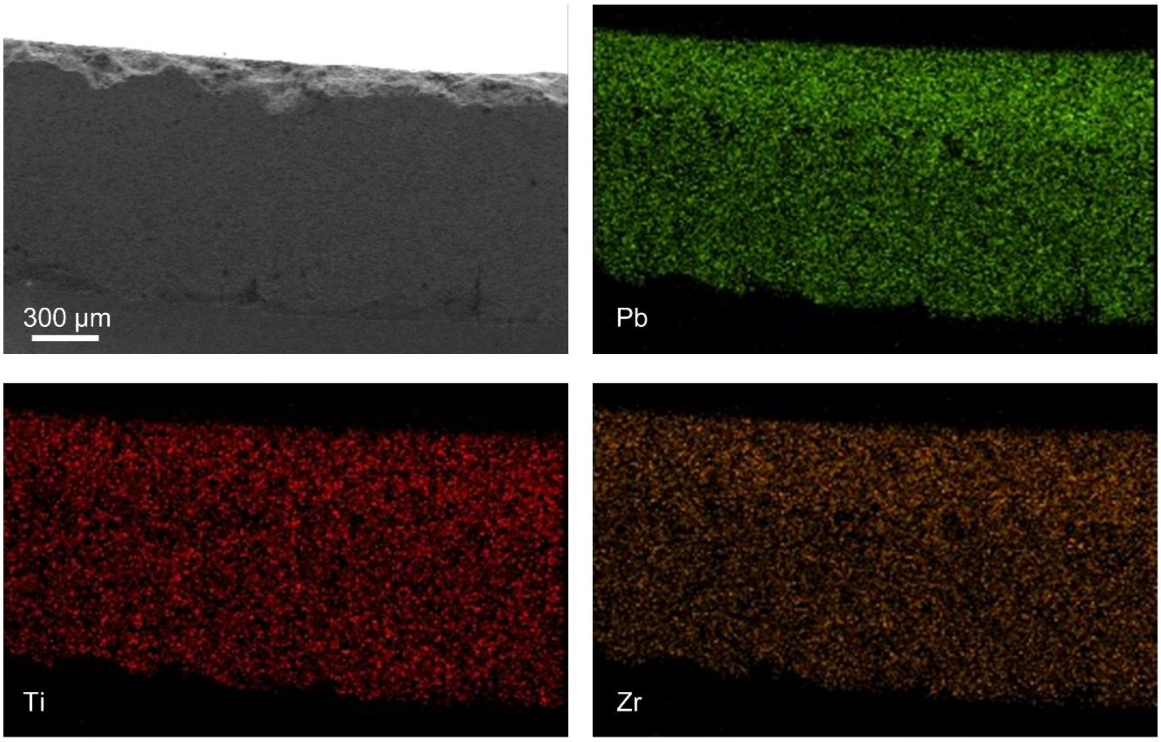

Supplementary Figure 2 | EDS mapping of a curved PZT ceramic. The elements of Pb, Zr and Ti exhibit a uniform distribution in the cross section of the curved PZT ceramic.

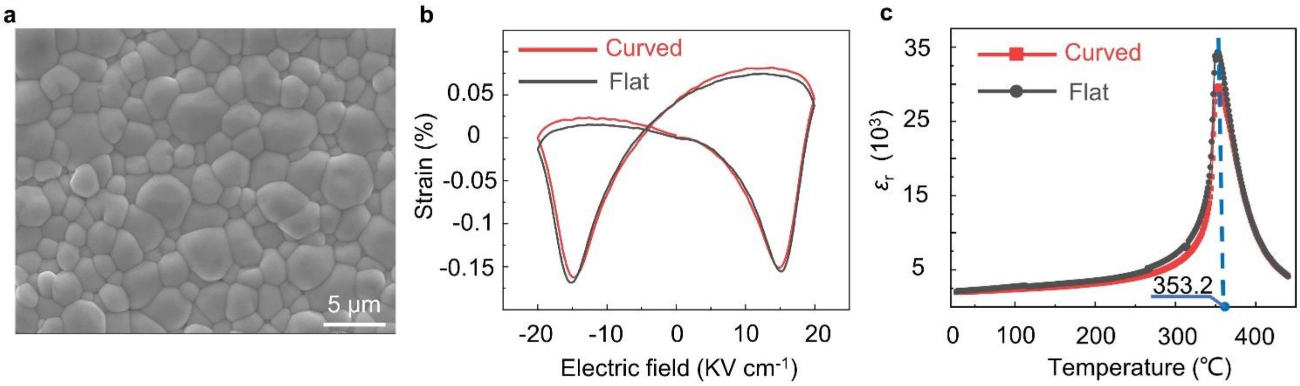

Supplementary Figure 3 | Material properties characterization of fabricating ceramics. a SEM image of the flat PZT ceramic. **b** Room-temperature *S*-*E* hysteresis loops of the curved and the flat PZT ceramic measured at 1 Hz. **c** Temperature dependence of relative dielectric constant ε_r of curved and flat PZT ceramics measured at frequency 1 kHz.

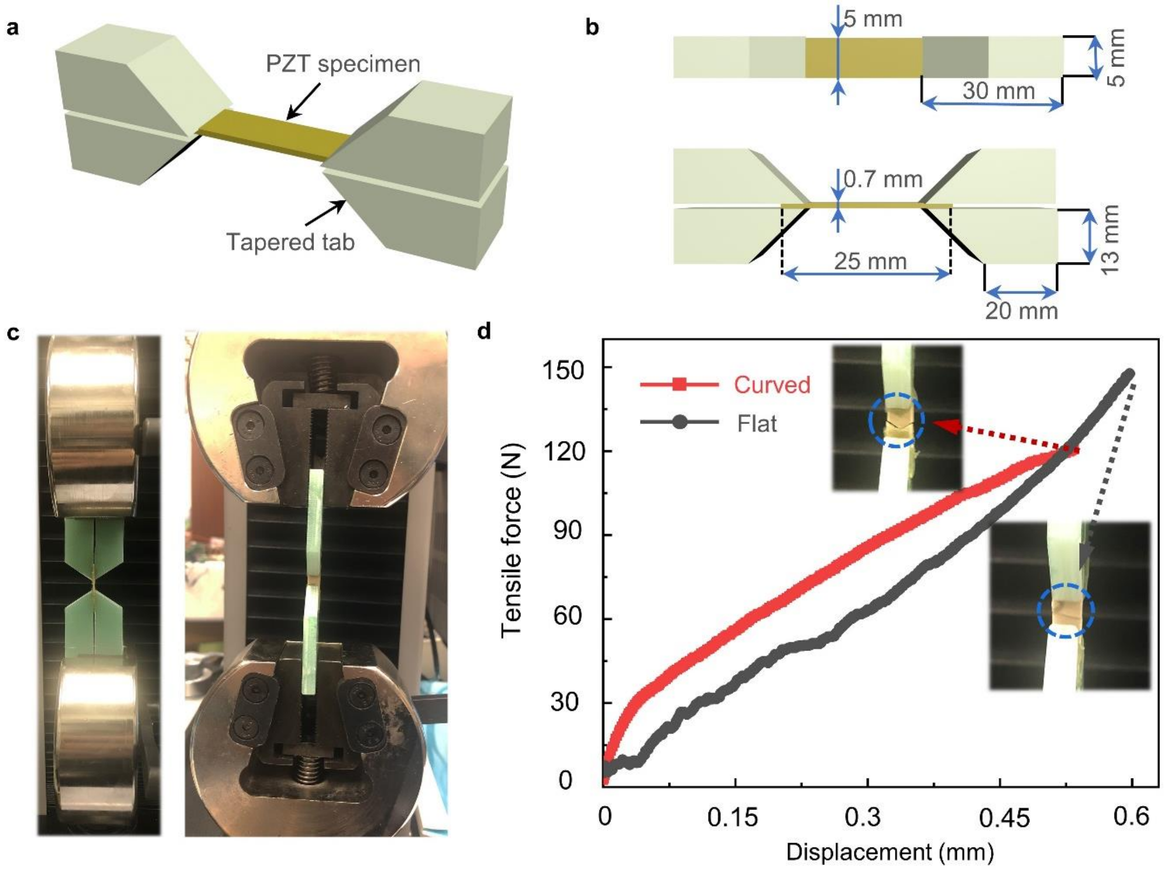

Supplementary Figure 4 | Tensile test for evaluating the mechanical quality of fabricated ceramics. a Schematic and **b** key parameters of the testing specimen and the tapered tabs. **c** Photographs of the testing system and clamped sample. **d** Tensile force-displacement curves of the curved and flat PZT ceramics measured with tensile loading (displacement) of 0.5 mm/min, where the corresponding fracture occurs on the middle of the samples (inset).

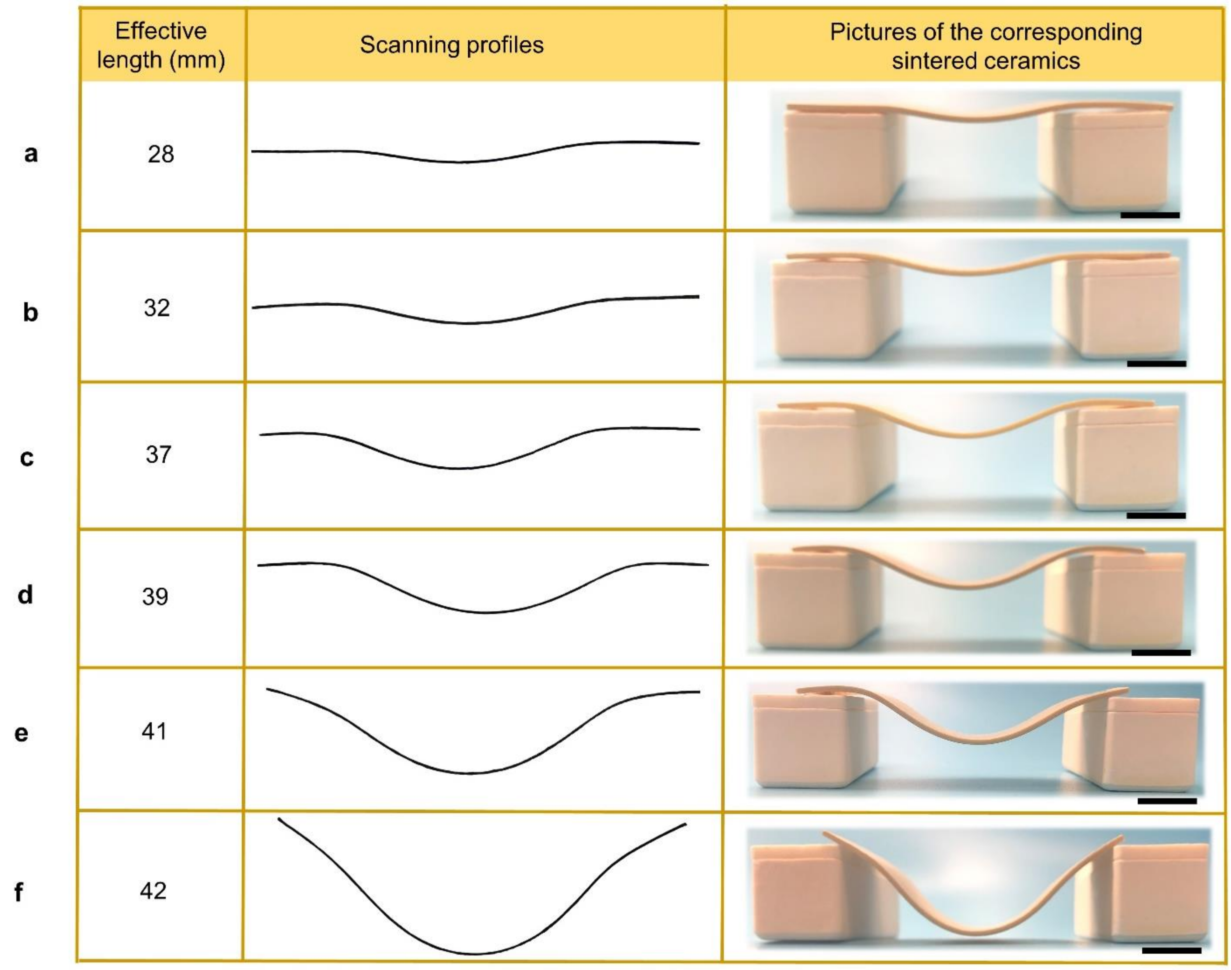

Supplementary Figure 5 | Corresponding photographs of the sintered PZT ceramics shown in Fig. 3c with effective lengths of **a** 28 mm **b** 32 mm **c** 37 mm **d** 39 mm **e** 41 mm **f** 42 mm. All scale bars are 10 mm in column 3.

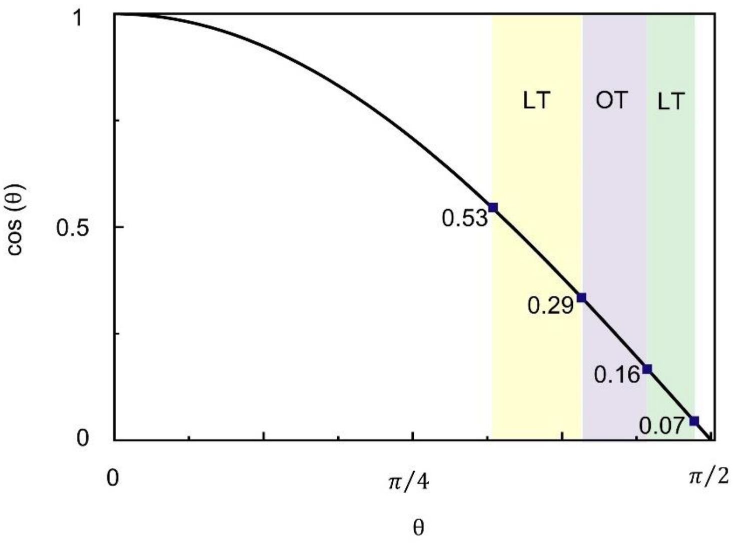

Supplementary Figure 6 | Values of $\cos\theta$ for the three tuning regions. The green, purple and yellow domains are corresponding to tunable region B, C and D shown in Fig. 3b.

Supplementary Table

Supplementary Table 1 | Material properties of the curved and flat PZT ceramics.

Parameters	Curved (GDS process)	Flat (Conventional)
Piezoelectric constant d_{33}	595 pC/N	600 pC/N
Relative free permittivity ε_{33}^{T} (1 kHz)	1988	2043
Dielectric loss tan δ	0.020	0.019
Currie temperature T_C	353.2 ºC	353.2 ºC
Tensile strength (unpoled)	36 MPa	43 MPa
Density ρ	7609 kg/m^3	7614 kg/m^3
